# Hereditary Persistence of Fetal Hemoglobin Presenting With 100% Fetal Hemoglobin and Recurrent Thrombotic Events

**DOI:** 10.7759/cureus.98434

**Published:** 2025-12-04

**Authors:** Garry G Lachhar, Salman Syed, Ali Abdullah, Alexandros Konstantinidis, Stephen Pentheros, Alfredo Torres

**Affiliations:** 1 Hematology and Oncology, Northwell Health, New York, USA; 2 Internal Medicine, Northwell Health, New York, USA; 3 Hematology and Oncology, New York Cancer and Blood Specialists, New York, USA; 4 Oncology, New York Cancer and Blood Specialists, New York, USA

**Keywords:** asian-indian deletion-inversion, beta-globin gene, fetal hemoglobin, hereditary persistence of fetal hemoglobin, thrombosis

## Abstract

Fetal hemoglobin (HbF) is the predominant hemoglobin during fetal development, typically replaced by adult hemoglobin after birth. Elevated HbF levels in adulthood are rare and often associated with genetic conditions such as hereditary persistence of fetal hemoglobin (HPFH) or hemoglobinopathies. We present the case of a 30-year-old Pakistani male with 100% HbF, recurrent thrombotic events, including splenic thrombosis and unprovoked pulmonary embolism, and chronic systemic symptoms. Despite extensive evaluation, including bone marrow biopsy, advanced imaging, and multidisciplinary consultations, establishing a definitive diagnosis remained challenging. Genetic testing revealed a homozygous Asian-Indian deletion-inversion in the *β-globin* cluster. The patient’s clinical course was complicated by persistent leukocytosis and compensated hemolysis. To our knowledge, this represents the first reported case in the literature of an adult with 100% HbF presenting with recurrent thrombotic complications. This case highlights the diagnostic complexity of rare hematological conditions and the potential association between 100% HbF and thrombotic complications, challenging the traditional view of HPFH as a benign condition.

## Introduction

Fetal hemoglobin (HbF), composed of two alpha and two gamma globin chains (α2γ2), is the primary oxygen carrier during fetal life. In healthy individuals, HbF levels decline rapidly after birth, comprising fewer than 1% of total hemoglobin by six months of age [1]. Hereditary persistence of fetal hemoglobin (HPFH) represents a group of genetic conditions characterized by continued *γ-globin* gene expression into adulthood, resulting in elevated HbF levels without significant hematological abnormalities [2].

HPFH can be classified into deletional and non-deletional forms. Deletional HPFH results from large deletions in the *β-globin* gene cluster that remove the δ and β genes while preserving or enhancing γ-gene expression. Non-deletional HPFH arises from point mutations in the *γ-globin* gene promoters that disrupt the normal developmental silencing of these genes [3]. The Asian-Indian deletion-inversion represents a unique form involving complex DNA rearrangement with both deletion and inversion of the *β-globin* cluster [4].

While HPFH is traditionally considered benign, emerging evidence suggests potential clinical implications. We present, to our knowledge, the first reported case in the literature of a young adult with 100% HbF complicated by recurrent thrombotic events despite anticoagulation therapy, highlighting the diagnostic and therapeutic complexities of this rare condition.

## Case presentation

A 30-year-old Pakistani male was referred to our hematology clinic for evaluation of persistent leukocytosis and constitutional symptoms. The patient presented with a several-month history of progressive fatigue, unintentional weight loss of 20 pounds, and postprandial left upper quadrant abdominal pain. His past medical history was significant for hepatitis C (treated and cured in Pakistan in 2015), splenic vein thrombosis requiring splenectomy (2018), and unprovoked pulmonary embolism (March 2020) that occurred one week after discontinuing anticoagulation.

Physical examination revealed pallor and mild scleral icterus. Initial laboratory evaluation findings are summarized in Table 1. Peripheral blood smear showed approximately 80% nucleated red blood cells with polychromasia, consistent with hemolysis. Hemoglobin electrophoresis revealed 100% HbF on multiple occasions.

**Table 1:** Initial laboratory findings. HbF = fetal hemoglobin

Laboratory test	Result	Reference range
Hemoglobin	11.2 g/dL	13.5–17.5 g/dL
White blood cell count	18,000/μL	4,500–11,000/μL
Platelet count	450,000/μL	150,000–400,000/μL
Indirect bilirubin	3.2 mg/dL	0.2–0.8 mg/dL
Lactate dehydrogenase	450 U/L	140–280 U/L
IgG4 level	180 mg/dL	<135 mg/dL
Hemoglobin electrophoresis	100% HbF	<1% HbF

Genetic testing using multiplex ligation-dependent probe amplification identified a homozygous Asian-Indian deletion-inversion in the *β-globin* cluster, confirming the diagnosis of HPFH. *JAK2 V617F* mutation was initially reported as positive, but was negative on repeat testing with bone marrow evaluation.

**Figure 1:**
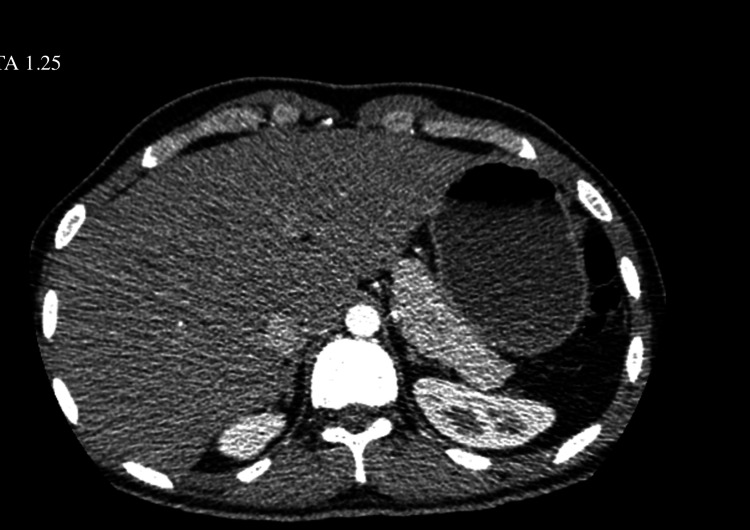
CT of the abdomen.

**Figure 2:**
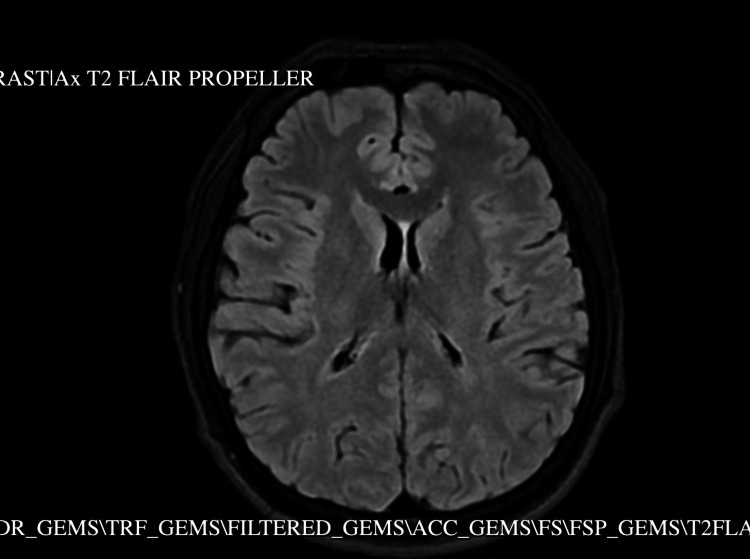
MRI/MRA of the brain.

Comprehensive imaging studies were performed to evaluate for occult malignancy given the patient’s recurrent thrombotic events. CT of the chest/abdomen/pelvis (Figure 1) and MRI/MRA (Figure 2) of the brain showed no significant abnormalities. Despite elevated IgG4 levels, autoimmune pancreatitis was considered, but the patient showed no response to a steroid trial.

**Figure 3:**
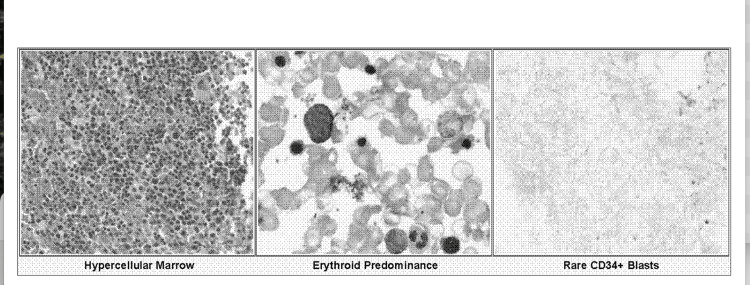
Bone marrow biopsy. Significant erythroid hyperplasia is seen with markedly hypercellular marrow for age, consistent with the patient’s history of deletion-inversion of the *β-globin* cluster with persistent fetal hemoglobin.

A bone marrow biopsy revealed a hypercellular marrow (90% cellularity) (Figure 3), with erythroid hyperplasia, but no evidence of malignancy or myeloproliferative neoplasm. Flow cytometry and paroxysmal nocturnal hemoglobinuria testing were negative.

Recurrent thrombotic events complicated the patient’s course despite anticoagulation therapy. While on rivaroxaban, he developed left upper extremity and right lower extremity deep vein thromboses. Subsequently, while on dabigatran, he experienced additional thrombotic events. Extensive hypercoagulability workup, including antiphospholipid antibodies, protein C, protein S, antithrombin III levels, Factor V Leiden, and prothrombin gene mutation, was unremarkable. An occult malignancy was extensively investigated through CT imaging, PET scan, and tumor markers, as well as a myeloproliferative neoplasm workup, all of which were negative. He is currently maintained on enoxaparin with close monitoring.

The patient continued experiencing approximately 20 daily episodes of weakness and dizziness, significantly impacting his quality of life. He also developed steatorrhea, managed with pancreatic enzyme supplementation. Despite comprehensive evaluation at multiple tertiary centers and referral to the Undiagnosed Diseases Network, no unifying diagnosis beyond HPFH with Asian-Indian deletion-inversion was established.

## Discussion

This case represents a rare presentation of homozygous Asian-Indian deletion-inversion resulting in 100% HbF production with significant clinical complications. To our knowledge, this is the first reported case in the literature of an adult patient with 100% HbF presenting with recurrent thrombotic complications. The uniqueness of this presentation lies not only in the complete absence of adult hemoglobin but also in the severe thrombotic phenotype despite anticoagulation therapy, which has not been previously described in HPFH.

The Asian-Indian inversion-deletion, first described by Fucharoen et al., involves a complex 48.5-kb deletion removing the δ and β genes combined with inversion of the remaining DNA sequence [5]. This mutation typically results in a thalassemia intermedia phenotype when inherited with another β-thalassemia allele, but homozygous cases are exceedingly rare [6].

The persistence of 100% HbF in our patient raises important pathophysiological considerations. While HbF has higher oxygen affinity than HbA, potentially causing relative tissue hypoxia, most individuals with HPFH remain asymptomatic [7]. However, our patient’s recurrent thrombotic events despite therapeutic anticoagulation (including failures on both rivaroxaban and dabigatran) suggest a profound prothrombotic state. Post-splenectomy status likely contributed to hypercoagulability, but cannot fully explain the refractory nature of his thromboses. The absence of identifiable hypercoagulable disorders or occult malignancy makes this case particularly intriguing. It suggests that extreme HbF elevation itself may confer thrombotic risk through mechanisms yet to be elucidated [8].

The multisystem involvement in this case, including chronic hemolysis and recurrent thrombotic events, presents a significant diagnostic challenge. While elevated IgG4 levels initially raised concern for an autoimmune process, the lack of steroid response and absence of other supportive findings made this unlikely. The chronic hemolysis evidenced by elevated indirect bilirubin and lactate dehydrogenase, along with compensated anemia, is consistent with the underlying hemoglobinopathy. However, the severity of thrombotic complications despite therapeutic anticoagulation suggests additional pathophysiological mechanisms beyond those typically seen in HPFH [9].

Recent studies have identified potential modifiers of HbF expression and clinical severity in β-hemoglobinopathies. Polymorphisms in *BCL11A*, *KLF1*, and the *β-globin* locus control region can influence HbF levels and disease phenotype [10]. These genetic modifiers may explain the variable clinical presentation among individuals with similar primary mutations and could guide future therapeutic approaches.

## Conclusions

This case illustrates the clinical complexity of homozygous Asian-Indian deletion-inversion presenting with 100% HbF and recurrent thrombotic complications despite anticoagulation therapy. To our knowledge, this represents the first reported case of an adult with 100% HbF manifesting with such a severe thrombotic phenotype. While HPFH is traditionally considered benign, our patient’s significant morbidity challenges this paradigm and suggests the need for increased surveillance in similar cases. The diagnostic odyssey highlights the importance of comprehensive genetic testing in patients with unexplained elevated HbF and the value of multidisciplinary collaboration in managing rare hematological conditions. Further research is needed to understand the thrombotic risk associated with extreme HbF elevation and to develop evidence-based management guidelines for these challenging cases.
